# Characterization of High-Temperature, Low-Temperature and Fatigue Performance of Phosphogypsum Warm-Mix Asphalt

**DOI:** 10.3390/ma19040713

**Published:** 2026-02-12

**Authors:** Xiaodong Jia, Li Ou, Hongzhou Zhu

**Affiliations:** 1 School of Urban Construction Engineering, Chongqing Open University, Chongqing 400052, China; jiaxd@cqtbi.edu.cn; 2 School of Civil Engineering, Chongqing Jiaotong University, Chongqing 400074, China; zhuhongzhouchina@cqjtu.edu.cn; 3 National & Local Joint Engineering Research Center of Transportation and Civil Engineering Materials, Chongqing 400074, China

**Keywords:** solid waste recycling, phosphogypsum, warm-mix asphalt, rheology

## Abstract

To explore the potential of phosphogypsum for resource utilization in asphalt pavements, this study evaluated its feasibility as a warm-mix asphalt (WMA) additive and investigated its influence on the rheological properties of asphalt binder. Phosphogypsum warm-mix asphalt was prepared by incorporating varying dosages of phosphogypsum warm-mix additive (PGWA) into both base asphalt and styrene–butadiene–styrene (SBS)-modified asphalt. The high-, medium-, and low-temperature performance of phosphogypsum warm-mix asphalt was evaluated using rheological tests. The results revealed that the complex modulus of PGWA-added base asphalt was higher than that of the base asphalt, with only minor changes in phase angle. The incorporation of the SBS modifier significantly enhanced the stiffness and elasticity of the asphalt binder. Compared with the control asphalt, PGWA-added asphalt exhibited lower creep strain and accumulated strain, higher creep recovery rates, and smaller non-recoverable compliance under the same stress level, indicating an improved resistance to high-temperature permanent deformation. PGWA increased the cumulative damage capacity and extended the fatigue life of the asphalt binder. Although the PGWA slightly reduced the low-temperature performance, the SBS modifier effectively compensated for this drawback. The Burgers model accurately captured the low-temperature rheological behavior of PGWA-added asphalt. Overall, PGWA-added asphalt demonstrated excellent rheological performance and high application potential, offering a promising pathway for the resource utilization of phosphogypsum and the development of sustainable, eco-friendly pavement materials.

## 1. Introduction

Warm-mix asphalt (WMA) technology is an asphalt pavement production method developed on the traditional hot-mix asphalt (HMA). Compared to conventional HMA, WMA achieves similar performance at lower mixing and construction temperatures, significantly reducing energy and greenhouse gas while effectively mitigating the thermal aging process of asphalt. Moreover, this technology improves the working environment on construction sites, reducing the potential health hazards posed by high temperatures to construction workers. Due to its notable energy-saving and environmental benefits, WMA technology has emerged as a research focus within green pavement engineering and the development of sustainable transportation infrastructure.

According to the cooling mechanisms, WMA technologies are typically classified into three types: organic additive, chemical additive, and foamed asphalt methods. Organic additives are typically composed of waxes and fatty amides. When incorporated into asphalt mixtures, they can effectively reduce the viscosity of asphalt and improve its lubricating properties at elevated temperatures, thereby enhancing the workability of the asphalt mixture [1,2]. During the cooling process, the crystallization and solidification of organic additives further strengthen the high-temperature performance of asphalt pavement [3]. Chemical additives were often used in conjunction with anti-stripping agents, emulsifiers, or surfactants to improve the workability of asphalt while enhancing other pavement performance characteristics [4,5]. Foamed asphalt technology can be achieved through either mechanical or material foaming. The former relies on high-pressure water injection to produce instantaneous expansion, while the latter utilizes the vaporization reactions of foaming materials during heating to achieve different construction effects [6,7].

At present, a wide variety of WMA additives have been developed. However, their relatively high cost limits the large-scale promotion and application of WMA technology in engineering practice. Therefore, developing novel WMA additives with high-performance and cost-effective characteristics is of great significance. Meanwhile, the steadily rising production of solid waste has emerged as a major environmental issue worldwide. Although governments have implemented various policies to promote the recycling and resource utilization of solid waste, the overall utilization rate remains low. Landfilling is still the dominant disposal method, which not only leads to resource wastage but also occupies large areas of land and imposes long-term pressure on the ecological environment. Solid wastes possess dual attributes as both pollutants and potential resources. If they can be utilized as WMA additives, it would not only enable the recycling and reduction in solid waste but also significantly lower the production cost of WMA. Recently, growing attention has been directed toward developing warm-mix asphalt additives from solid waste materials, focusing on reducing costs and enhancing value.

Previous studies have shown that geopolymers, composed of Si–O and Al–O tetrahedral structures, possess abundant internal pores capable of accommodating water molecules. Warm-mix additives prepared from raw materials such as fly ash and coal gangue can not only significantly reduce the production cost of WMA but also achieve remarkable energy-saving and emission-reduction effects [8,9]. In addition, waste polyethylene terephthalate materials can be transformed into water-containing foamed asphalt additives through ammonolysis, and the resulting asphalt exhibits road performance comparable to that of conventional HMA [10]. Through catalytic pyrolysis in a pressurized reactor, waste linear low-density polyethylene can be converted into polyethylene wax for the preparation of WMA, providing a sustainable pathway for resource utilization [11]. Similarly, waste polypropylene can be converted into high-performance warm-mix asphalt modifiers via thermochemical and mechanochemical methods, significantly enhancing the high-temperature performance, low-temperature cracking resistance, and fatigue durability [12,13]. Zeolites synthesized from sewage sludge ash have also been employed as WMA additives, showing engineering performance comparable to that of traditional HMA, with notable environmental and economic benefits [14]. The reuse of biomass resources has also become a research hotspot. For instance, palm methyl ester, a phase-change component derived from waste edible oil through transesterification and fractional distillation in biodiesel production, has been utilized as a warm-mix asphalt additive [15,16]. A composite warm-mix additive produced by incorporating waste cooking oil with discarded low-density polyethylene particles can substantially lower asphalt viscosity while simultaneously enhancing its high-temperature stability and fatigue resistance [17]. These studies demonstrate that solid waste-derived materials have shown great potential as warm-mix asphalt additives, offering both engineering feasibility and environmental benefits.

Among various industrial solid wastes, phosphogypsum has attracted increasing attention due to its enormous production volume and severe environmental risks. Phosphogypsum is an industrial by-product generated during the wet-process production of phosphoric acid, with approximately 4–5 t of phosphogypsum produced per ton of phosphoric acid [18]. The origin and physicochemical properties of phosphogypsum are strongly influenced by the type of phosphate rock, phosphoric acid production conditions and efficiency, aging time, and the chemical reagents used, resulting in significant variations in crystal morphology, particle size distribution, and impurity composition [19]. Phosphogypsum mainly consists of calcium sulfate dihydrate (CaSO_4_·2H_2_O), accounting for about 90%, with an adherent water content of 10–30% and particle sizes typically ranging from 5 to 150 μm. Phosphogypsum contains minor amounts of impurities such as SiO_2_, Al_2_O_3_, Fe_2_O_3_, CaO, MgO, and F [20]. Improper storage or disposal of phosphogypsum may lead to environmental risks including water and soil contamination, dust dispersion, and extensive land occupation [21]. In some countries, phosphogypsum has even been directly discharged into the ocean, potentially facilitating the transfer of toxic and hazardous elements into marine ecosystems [22]. The resource utilization of phosphogypsum therefore represents a promising pathway for mitigating environmental pollution, reducing carbon emissions, and promoting the sustainable development of related industries.

However, in the preparation of warm-mix additives from solid waste materials, including phosphogypsum, existing studies have not fully considered the compositional variations and performance fluctuations inherent in solid waste materials. To achieve the stable application of these materials in warm-mix asphalt technology, it is essential to ensure the consistency of their composition and the reproducibility of their performance, thus ensuring the reliability and engineering applicability of the warm-mix effect. Studies have shown that through appropriate pretreatment processes, impurities can be effectively removed from phosphogypsum, resulting in phosphogypsum warm-mix additive (PGWA) with a purity greater than 99% [23]. This result suggests that employing phosphogypsum as a WMA additive not only offers a viable pathway for its utilization but also helps reduce the environmental and management burdens associated with phosphogypsum disposal. Nevertheless, existing studies have yet to investigate the high-temperature deformation resistance, low-temperature cracking performance, and fatigue life of asphalt incorporating PGWA.

## 2. Objective and Scope

**Figure 1:**
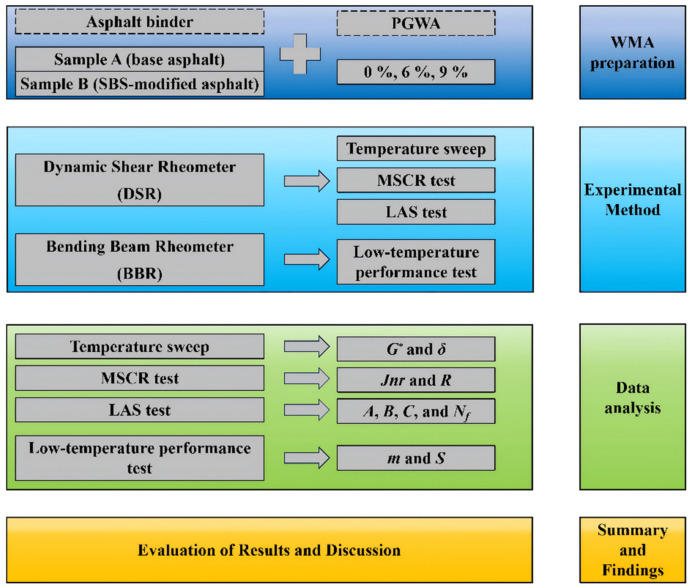
The overall research procedure of this study.

This study aims to systematically evaluate the effect of different PGWA contents (0%, 6%, and 9%) on the rheological properties of two types of asphalt binder (base asphalt and SBS-modified asphalt). Dynamic shear oscillatory tests, multiple stress creep and recovery (MSCR), and linear amplitude sweep (LAS) experiments were performed using a dynamic shear rheometer (DSR) to characterize the complex shear modulus, phase angle, creep recovery, non-recoverable creep compliance, and fatigue life of asphalt containing PGWA. In addition, the stiffness modulus and low-temperature creep rate were determined with a bending beam rheometer (BBR), and the low-temperature performance of PGWA-added asphalt was modeled and comprehensively evaluated using the Burgers model. The overall research procedure is illustrated in Figure 1.

## 3. Materials and Methods

### 3.1. Raw Materials

In this study, 70# base asphalt and SBS-modified asphalt were selected, both produced by Fujian Petrochemical Co., Ltd. (Quanzhou, China). The various performance indicators of asphalt binder were tested according to the Standard of JTG 3410-2025 [24]. The test results are shown in Table 1, and all indicators met the required specifications. Phosphogypsum was sourced from Hubei Yihua Fertilizer Co., Ltd. (Yichang, China), and it passed testing via the Three Gorges Public Inspection and Testing Center before being released. PGWA is prepared by the atmospheric acidification method, and the detailed process can be found in reference [23].

### 3.2. Preparation of Warm-Mix Asphalt

In this study, three PGWA contents (0%, 6%, and 9% by asphalt mass) were designed for the preparation of WMA. First, the asphalt and PGWA were measured according to the specified proportions. The 70# and SBS-modified asphalts were heated in an oil bath at 150 °C and 175 °C, respectively. PGWA was then introduced into the molten asphalt, and the blend was mechanically stirred at 1200 rpm for 10 min to achieve a uniform dispersion of PGWA within the binder.

### 3.3. Dynamic Shear Oscillatory Test

The dynamic shear oscillatory tests of PGWA-added asphalt were conducted using a TA DSR. The temperature sweep was conducted from 25 °C to 75 °C. The applied strain was determined to be 10% through amplitude sweep testing, ensuring that all samples were evaluated within the linear viscoelastic region.

### 3.4. MSCR Test

The MSCR test was employed to analyze the variations in creep recovery rate (*R*) and non-recoverable compliance (*J*nr) of PGWA-added asphalt under high-temperature conditions. The *R* value indicates the degree to which the asphalt sample recovers its original position after the load is removed [25]. The *J*nr value corresponds to the residual strain remaining in the asphalt after experiencing both linear and nonlinear viscoelastic behavior under higher temperature and stress conditions. Compared with the conventional rutting factor, the *J*nr value more accurately reflects the rutting characteristics of asphalt because it accounts for the viscoelastic behavior under actual loading conditions [26]. The parameters *R* and *J*nr at different stress levels were calculated using Equations (1) and (2).

(1) $$R=\frac{\varepsilon_{c}-\varepsilon_{r}}{\varepsilon_{c}-\varepsilon_{0}}$$

(2) $$J_{nr}=\frac{\varepsilon_{r}-\varepsilon_{0}}{\tau }$$

Here, $\varepsilon_{c}$ is the peak strain, $\varepsilon_{r}$ represents the unrecovered strain, $\varepsilon_{0}$ denotes the initial strain, and τ indicates the applied stress level.

### 3.5. LAS Test

The LAS test was performed at a temperature of 25 °C. According to AASHTO TP101-14 [27], the LAS test begins with a specific frequency sweep. Subsequently, the amplitude sweep was performed at a frequency of 10 Hz. The fatigue relationship is represented by Equation (3).

(3) $$N_{f}=A{\left(\gamma_{max}\right)}^{-B}$$

Here, *A* and *B* are the fatigue model parameters, $N_{f}$ indicates the fatigue life, and $\gamma_{max}$ denotes the estimated maximum pavement strain (%).

### 3.6. Low-Temperature Bending Rheological Properties

The low-temperature performance of PGWA-added asphalt was tested using a servo-controlled thermoelectric BBR manufactured by Matest-Pavetest, Italy. Anhydrous ethanol was used as the testing bath medium, equipped with a built-in magnetic stirring rotor to maintain temperature uniformity. The BBR test was employed to determine the bending creep stiffness modulus (*S*) of asphalt samples. Based on the power-law function, the stiffness rate (*m*-value) was calculated to analyze the variation in *S* and *m* with temperature. Furthermore, the Burgers viscoelastic constitutive model was applied to fit the BBR test results of PGWA-added asphalt, thereby quantitatively characterizing its low-temperature cracking resistance.

#### 3.6.1. Creep Behavior Analysis

The BBR test was conducted to simulate the performance evolution of PGWA-added asphalt under low-temperature conditions, in order to evaluate its creep stiffness and stress relaxation behavior. Based on the specimen dimensions and the automatically recorded load and deflection data, the stiffness and stiffness variation rate were computed according to Equations (4) and (5), respectively.

(4) $$S_{m}(t)=PL^{3}/4bh^{3}\delta (t)$$

(5) m(t)=|dlog[S(t)]/dlog(t)|

Here, $S_{m}(t)$ is the bending creep stiffness and m(t) is the rate of change in bending creep stiffness.

#### 3.6.2. Burgers Model

The Burgers model, which consists of a Maxwell model and a Kelvin model, can be used to evaluate energy dissipation and storage in viscoelastic materials [28,29,30,31]. The constitutive second-order differential equation of the Burgers model describes the variation in stress and strain with time t, as expressed in Equations (6)–(10).

(6) $$K_{1}\frac{d^{2}\sigma }{dt^{2}}+K_{2}\frac{d\sigma }{dt}+\sigma =K_{3}\frac{d^{2}\varepsilon }{dt}+K_{4}\frac{d\varepsilon }{dt}$$

(7) $$K_{1}=\frac{\eta_{1}\eta_{2}}{E_{1}E_{2}}$$

(8) $$K_{2}=\frac{\eta_{1}}{E_{1}}+\frac{\eta_{2}}{E_{2}}+\frac{\eta_{1}}{E_{2}}$$

(9) $$K_{3}=\frac{\eta_{1}\eta_{2}}{E_{2}}$$

(10) $$K_{4}=\eta_{1}$$

Here, *E*_1_ represents the instantaneous elastic modulus, *E*_2_ denotes the delayed elastic modulus, *η*_1_ is the instantaneous viscosity coefficient, and *η*_2_ corresponds to the delayed viscosity coefficient.

**Figure 2:**
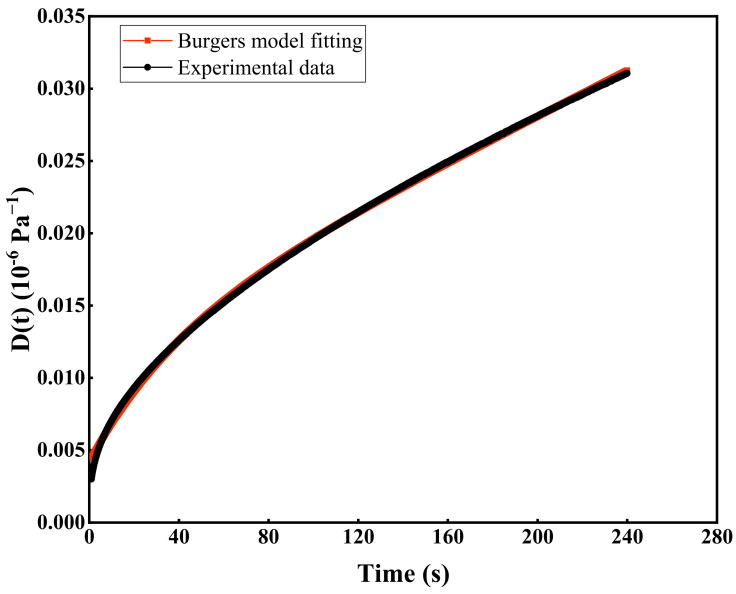
Schematic diagram of Burgers model fitting.

By fitting the creep data obtained from the BBR test, the parameters of the Burgers model can be determined. A schematic of the fitting results is shown in Figure 2.

To further analyze the low-temperature characteristics of phosphogypsum warm-mix asphalt, the comprehensive compliance parameter (*J*_C_) was introduced to evaluate the low-temperature viscoelastic behavior [32]. The *J*_C_ value is given in Equations (11)–(14).

(11) $$J_{c}=\frac{1}{J_{V}\left(1-\frac{J_{E}+J_{DE}}{J_{E}+J_{DE}+J_{V}}\right)}$$

(12) $$J_{E}=1/E_{1}$$

(13) $$J_{DE}=1/E_{2}\left(1-e^{-\frac{tE_{2}}{\eta_{2}}}\right)$$

(14) $$J_{V}=\frac{t}{\eta_{1}}$$

Here, *J_E_* is the instantaneous elastic compliance, *J_DE_* is the delayed elastic compliance, *t* is the creep time, and *J_V_* is the viscous flow compliance.

## 4. Results and Discussion

### 4.1. Complex Modulus and Phase Angle of PGWA-Added Asphalt

**Figure 3:**
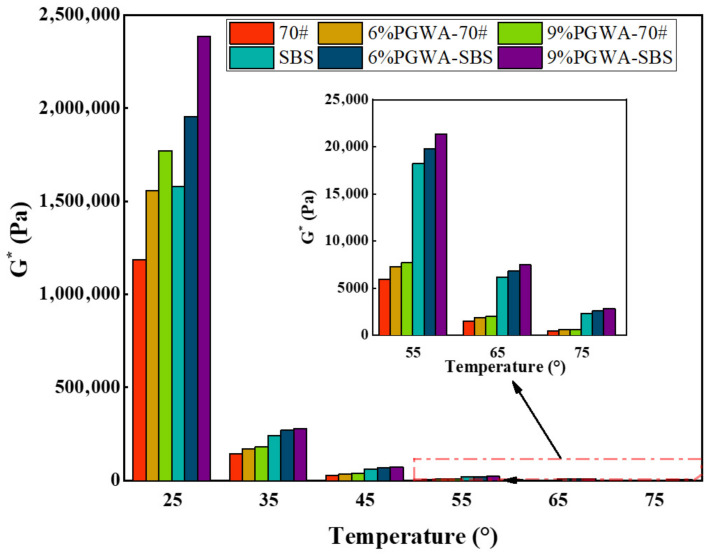
Complex shear modulus of PGWA-added asphalt.

The variation in complex shear modulus of PGWA-added asphalt with temperature is shown in Figure 3. Within the temperature range of 25–75 °C, the complex shear modulus of PGWA-added SBS-modified asphalt is significantly higher than that of PGWA-added base asphalt, with the difference becoming more pronounced at higher temperatures. At 45 °C, the complex modulus of PGWA-added base asphalt increases from 28.4 kPa (control) to 35.8 kPa (9% PGWA), corresponding to an improvement of approximately 26%, while 9%PGWA-added SBS-modified asphalt reaches 72.2 kPa. The introduction of PGWA generally resulted in an increase in high-temperature stiffness and an improvement in rutting resistance [33].

**Figure 4:**
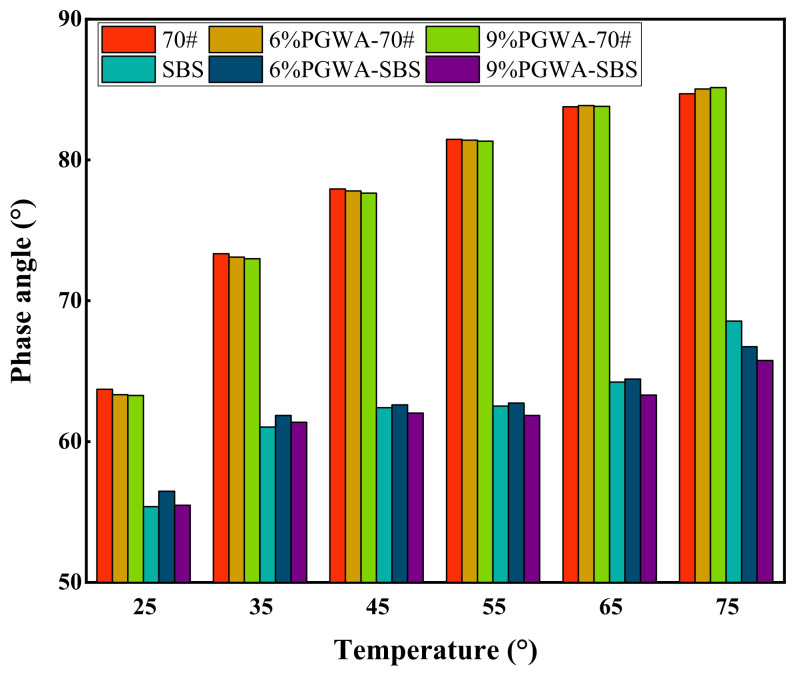
Phase angle of PGWA-added asphalt.

The variation in phase angle of PGWA-added asphalt with temperature is shown in Figure 4. Under the same temperature and asphalt type, the differences in phase angle among samples with different PGWA contents are relatively small, and their overall trends are consistent. In contrast, the incorporation of SBS modifier significantly reduces the phase angle of PGWA-added asphalt, enabling the material to maintain a stronger elastic response over a wider temperature range and thereby enhancing its high-temperature deformation resistance and structural stability. As the temperature increases, the phase angle of PGWA-added base asphalt significantly increases, indicating a transition in its viscoelastic behavior from an elasticity-dominated response to a viscosity-dominated response [34].

### 4.2. High-Temperature Performance of PGWA-Added Asphalt

**Figure 5:**
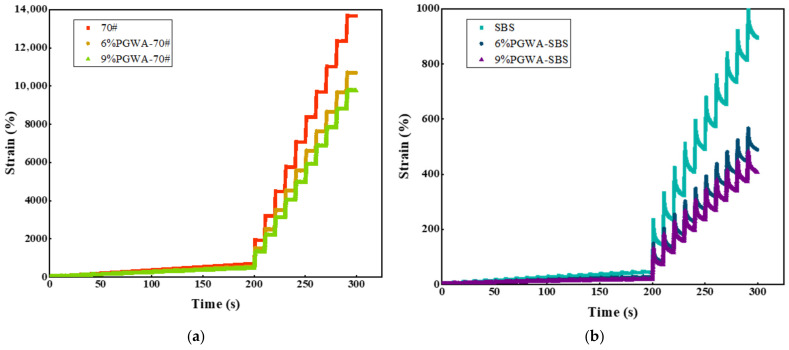
Time–strain relationship of PGWA-added asphalt: (**a**) 70# base asphalt; (**b**) SBS-modified asphalt.

The creep recovery curve of PGWA-added asphalt at 60 °C is shown in Figure 5. Distinct variations were observed among the strain–time curves of different PGWA-added asphalt binders. As illustrated in Figure 5, the recovery curve of PGWA-added base asphalt is relatively gentle and step-shaped, indicating that the deformation under loading is predominantly non-recoverable permanent deformation. This behavior reflects a lower elastic component and a higher viscous component, suggesting that the material approaches a viscous flow state at high temperatures. In contrast, PGWA-added SBS-modified asphalt exhibits smaller deformation, and its curve drops rapidly during the unloading stage, indicating a higher proportion of recoverable deformation after loading. This demonstrates a more pronounced elastic response and excellent high-temperature stability. For the given type of asphalt binder, increasing the PGWA content leads to a decrease in accumulated strain.

**Figure 6:**
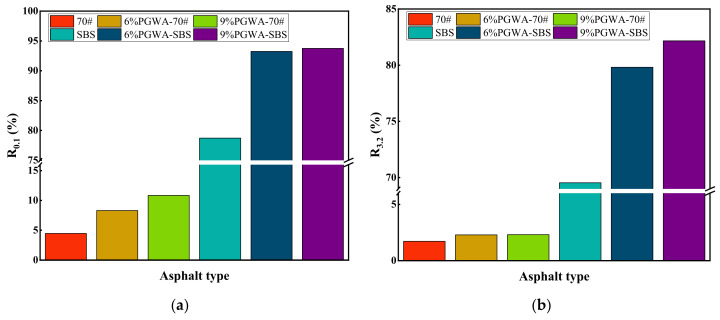
Creep recovery rate of PGWA-added asphalt: (**a**) 0.1 kPa; (**b**) 3.2 kPa.

The R values of PGWA-added asphalt under the stress level of 0.1 kPa are shown in Figure 6a. PGWA enhances the elastic deformation recovery capacity of asphalt after loading at elevated temperatures. At 60 °C and 0.1 kPa, the R value of base asphalt increases from 4.5% (control) to 10.8% (9% PGWA), corresponding to an improvement of approximately 140%. The creep recovery behavior of PGWA-added SBS-modified asphalt is largely governed by the extent of polymer dispersion within the binder and its capacity to establish a dominant structural network.

**Figure 7:**
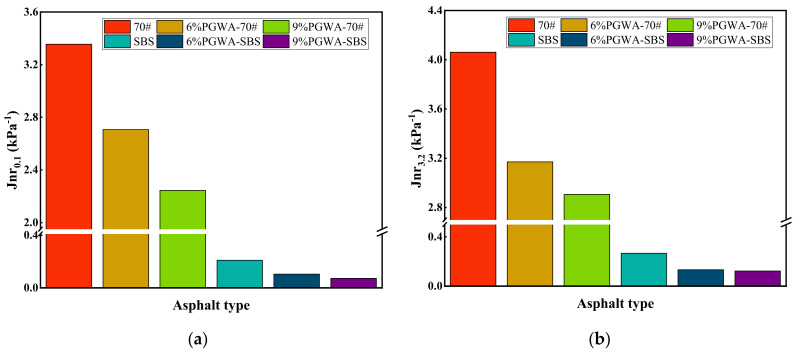
Non-recoverable compliance of PGWA-added asphalt: (**a**) 0.1 kPa; (**b**) 3.2 kPa.

The non-recoverable compliance of PGWA-added asphalt at 0.1 kPa is shown in Figure 7a. A lower Jnr value indicates that the asphalt experiences less permanent deformation under load, meaning that its resistance to high-temperature flow and plastic deformation is enhanced [35]. Compared to HMA, PGWA-added asphalt reduces the non-recoverable compliance, suggesting that PGWA decreases irreversible deformation and increases elastic recovery capacity.

The non-recoverable compliance at 3.2 kPa is shown in Figure 7b. As the applied stress increases at a constant temperature, the non-recoverable compliance also increases. With the incorporation of PGWA, the non-recoverable compliance of the asphalt is reduced, demonstrating that PGWA more effectively improves the resistance of asphalt to permanent deformation. At 60 °C and 3.2 kPa, the Jnr value of the base asphalt decreases from 4.1 kPa^−1^ for the control sample to 2.9 kPa^−1^ for the 9% PGWA-added asphalt, confirming that PGWA maintains rutting resistance enhancement even under high stress loading. PGWA-added SBS-modified asphalt exhibits lower non-recoverable compliance, further validating its excellent high-temperature performance.

### 4.3. Fatigue Performance of PGWA-Added Asphalt

**Figure 8:**
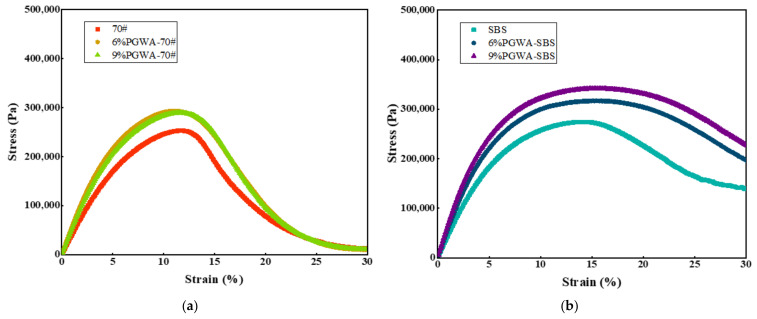
Stress–strain curve of PGWA-added asphalt: (**a**) 70# base asphalt; (**b**) SBS-modified asphalt.

The stress–strain curve of PGWA-added asphalt is shown in Figure 8. Once the strain exceeds the yield strain, the shear stress gradually decreases, indicating that the asphalt material begins to experience fatigue failure. The difference in yield strain among the various PGWA-added asphalt samples is relatively small. Compared to conventional HMA, the PGWA-added asphalt with SBS modifier exhibits a more gradual stress–strain curve upon reaching the peak stress, suggesting a longer strain accumulation before failure, thus demonstrating better ductility and improved fatigue crack resistance.

**Figure 9:**
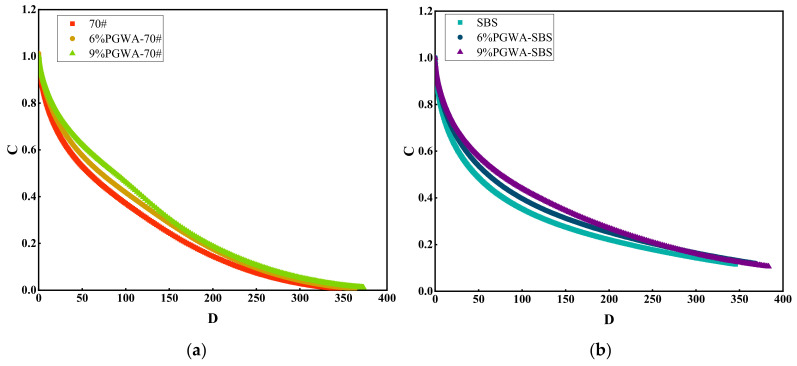
Cumulative damage curve of PGWA-added asphalt: (**a**) 70# base asphalt; (**b**) SBS-modified asphalt.

The cumulative damage curve of PGWA-added asphalt is shown in Figure 9. The integrity index (C) value of one indicates that the asphalt remains undamaged, while a decrease in C value toward zero reflects the accumulation of fatigue damage and, ultimately, complete failure [36]. When the C value reaches its critical threshold, the HMA without PGWA shows the greatest cumulative damage, whereas the PGWA-added asphalt exhibits the least. Based on the integrity index as the failure criterion, it can be inferred that incorporating PGWA effectively extends the fatigue life of the asphalt. As damage increases, the integrity index of the PGWA-added base asphalt approaches zero, displaying evident fatigue degradation characteristics. In contrast, the integrity index of the PGWA-added SBS-modified asphalt remains above 0.1, indicating that the SBS modifier also positively influences the fatigue performance of the asphalt. The damage characteristic curve illustrates the damage process experienced by PGWA-added asphalt under load, but additional parameters should be considered for a comprehensive assessment of the fatigue performance of asphalt [37].

**Figure 10:**
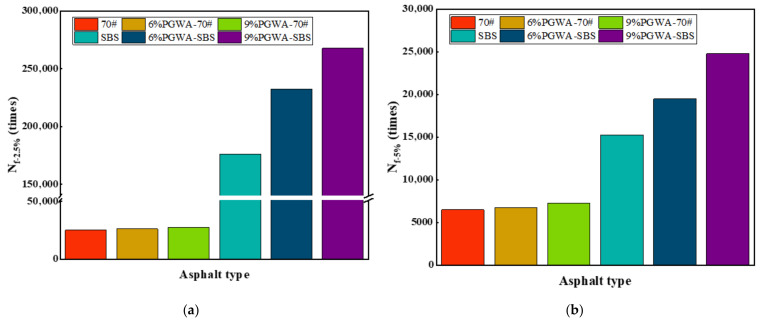
Fatigue life of PGWA-added asphalt: (**a**) 2.5% strain level; (**b**) 5% strain level.

Two strain levels of 2.5% and 5% were selected to evaluate the fatigue performance of adding PGWA asphalt, with 2.5% corresponding to light traffic and 5% to heavy traffic conditions [38,39]. From Figure 10, the fatigue life of the asphalt differs substantially between the two strain levels, exhibiting variations by orders of magnitude. At a strain level of 2.5%, the fatigue life of SBS-modified asphalt increases from 176,321 cycles (control) to 267,808 cycles (9% PGWA), corresponding to an improvement of approximately 52%. The improvement in fatigue life resulting from the addition of the PGWA can be attributed to two primary factors. First, PGWA exhibits enhanced fatigue resistance under cyclic loading, which may be associated with the morphology of PGWA particles [23]. Second, PGWA reduces the construction temperature, thereby mitigating asphalt aging and allowing the binder to retain higher ductility and viscoelastic recovery capacity. These two mechanisms work synergistically to slow down the fatigue damage evolution process, significantly extending the fatigue life of the asphalt.

### 4.4. Low-Temperature Bending Rheological Property

#### 4.4.1. Creep Stiffness and Creep Rate

**Figure 11:**
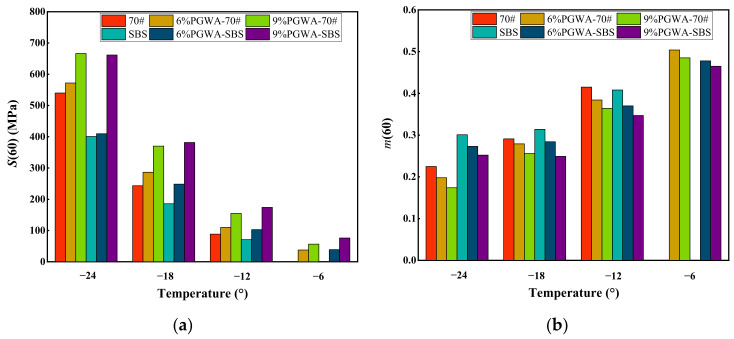
Low-temperature bending rheological properties of PGWA-added asphalt: (**a**) creep stiffness; (**b**) rate of change in stiffness.

A higher stiffness change rate (*m*) calculated from the BBR test indicates better low-temperature performance of asphalt, while a higher creep stiffness (*S*) corresponds to poorer low-temperature cracking resistance [40]. The low-temperature bending tests conducted at −6 °C, −12 °C, −18 °C, and −24 °C for 60 s are shown in Figure 11. With the increase in PGWA content, the creep stiffness of the PGWA-added asphalt gradually increases, whereas the stiffness change rate decreases. At −12 °C, *S*(60) increases from 88.8 MPa to 155 MPa, while *m*(60) decreases from 0.42 to 0.38, with the PGWA-added base asphalt content increasing from 0 to 9%. This suggests that the incorporation of the PGWA causes the asphalt system to exhibit higher stiffness and greater brittleness at low temperatures, leading to reduced deformation capacity and a slower stress relaxation rate, thereby weakening its low-temperature cracking resistance. This behavior can be attributed to the inherent rigidity of the PGWA particles and their constraint on the molecular chain mobility within the asphalt binder.

#### 4.4.2. Low-Temperature Performance Analysis Based on Burgers Model

The four viscoelastic parameters in the Burgers model are *E*_1_, *E*_2_, *η*_1_, and *η*_2_, while the relaxation time can be calculated using λ = *η*_1_/*E*_1_. The fitting results of each parameter are presented in Table 2. At the temperature of −6 °C, the deflection of the asphalt beam exceeds the measurement range of BBR, so there is no available data in the group without adding PGWA at this temperature condition.

The Burgers model fitting parameters for the asphalt containing PGWA are presented in Table 2. All correlation coefficients of the fitted parameters are greater than 0.97, indicating that the Burgers model provides highly reliable and accurate fitting results. As the temperature decreases, *E*_1_, *E*_2_, *η*_1_, and *η*_2_ all exhibit an increasing trend, suggesting that the viscoelastic parameters of PGWA increase with decreasing temperature. With the incorporation of the PGWA, the viscoelastic parameters show a trend similar to that observed with temperature reduction. However, the addition of the SBS modifier leads to the opposite behavior. Among the fitted parameters, *E*_1_ and *E*_2_ are significantly smaller than *η*_1_ and *η*_2_, indicating that the viscous component predominates over the elastic component in the viscoelastic behavior of PGWA-added asphalt.

The relaxation coefficient (λ) represents the ability of PGWA-added asphalt to dissipate stress under load. A lower λ value indicates faster stress relaxation and better low-temperature performance. As the PGWA content increases, the λ value also rises, implying that the additive negatively impacts the low-temperature behavior of asphalt. This behavior may be related to the interaction between PGWA and asphalt, which could influence the distribution and mobility of the binder within the WMA, thereby affecting its low-temperature flexibility [41].

The reduction in temperature lowers the mobility of molecular functional groups in the asphalt, thereby reducing its deformation capacity and increasing the risk of low-temperature cracking. The SBS modifier mitigates energy dissipation by forming a network structure within the asphalt, enhancing its viscoelasticity and improving its low-temperature behavior. Furthermore, the relaxation time increases with higher PGWA content, implying that the additive promotes energy dissipation and diminishes the viscoelastic capacity of materials.

The flexibility coefficient (*J_C_*) of PGWA-added asphalt is shown in Table 3. The *J_C_* value indicates the relative proportions of the asphalt’s viscous and elastic components. A higher *J_C_* value signifies a larger elastic fraction and a smaller viscous fraction. At low temperatures, a lower *J_C_* value typically correlates with improved resistance to low-temperature cracking. As presented in Table 3, the values of *J_E_*, *J_V_*, and *J*_DE_ decrease with increasing PGWA content, while the *J*_C_ value gradually increases. Additionally, the *J*_C_ value rises as the temperature decreases, suggesting that the low-temperature performance of PGWA-added asphalt worsens at lower temperatures. At a given temperature, higher PGWA content results in increased *J*_C_ values, while the addition of SBS modifier reduces them. This indicates that PGWA diminishes the low-temperature performance of asphalt, whereas the SBS modifier enhances it, aligning with the earlier observations.

In summary, the low-temperature performance parameters of PGWA-added asphalt obtained from the Burgers model fitting show good consistency between the λ and *J_C_* indicators. Both the PGWA content and the test temperature exhibit positive correlations with λ and *J_C_*, indicating that PGWA has a detrimental effect on the low-temperature performance of asphalt. In contrast, the SBS modifier shows negative correlations with λ and *J_C_*, demonstrating its effectiveness in improving the viscoelastic properties of PGWA-added asphalt and enhancing its stress relaxation and deformation recovery capabilities.

## 5. Conclusions

In this study, the rheological and viscoelastic properties of PGWA-added asphalt were systematically evaluated through high- and low-temperature rheological indices, and the influence of the PGWA on the fatigue performance of asphalt was investigated. The main conclusions are as follows:

(1) The complex modulus of PGWA-added base asphalt is consistently higher than that of base asphalt, while the difference in phase angle remains small. The SBS modifier provides a more pronounced improvement in high-temperature performance than the PGWA, leading to a substantial increase in complex modulus. Meanwhile, the PGWA maintains a relatively stable proportion between the viscous and elastic components of the asphalt.
(2) Under the same stress conditions, the creep strain and accumulated strain of PGWA-added asphalt are lower than those of HMA. The creep recovery rate of PGWA-added asphalt increases, while the non-recoverable compliance decreases, indicating that the PGWA contributes to improving the rutting resistance of asphalt.
(3) With the addition of the PGWA, the yield stress of the asphalt gradually increases, while the variation in yield strain remains relatively small. Among the evaluated cumulative damage parameters, the base asphalt exhibits the lowest level of accumulated damage. Incorporating the PGWA enhances the ability of asphalt to withstand cumulative damage and improves the fatigue life.
(4) The creep stiffness of PGWA-added asphalt increases with higher additive content, while the creep rate decreases, indicating that the PGWA reduces the low-temperature cracking resistance of asphalt. The relaxation time calculated based on the Burgers model shows a variation trend consistent with that of the compliance parameters, confirming the applicability of the model for evaluating the low-temperature behavior of PGWA-added asphalt.

In summary, PGWA is a promising WMA with significant value, demonstrating potential practical applications in pavement design and maintenance. It is particularly beneficial in regions where high-temperature stability and fatigue resistance are critical, offering a solution to alleviate the environmental pressures and handling challenges associated with road management. However, it is important to note that the findings of this study are based primarily on laboratory experiments. Future research should focus on further exploring the micro-mechanisms of action, aging behavior, and energy-saving and emission-reduction effects, and validating these results through field pavement trials.

## Figures and Tables

**Table 1:** The physical properties of asphalt binder.

Properties	Penetration /0.1 mm	Softening Point /°C	Ductility/cm		RTFO (163 °C, 85 min)			
			5 °C	15 °C	Quality Change /%	Residual Penetration Ration /%	Residual Ductility /cm	
							5 °C	10 °C
Base asphalt (70#)	68.1	48.8		>100	−0.06	64.5		8.4
SBS-modified asphalt	55	68	26		0.5	71	16	

**Table 2:** Burgers model parameters of PGWA-added asphalt.

Types of Asphalt	Test Temperature (℃)	*E*_1_ (MPa)	*η*_1_ (MPa·s)	*E*_2_ (MPa)	*η*_2_ (MPa·s)	λ	*R* ^2^
6%PGWA-SBS	−6	193.39	8731.96	95.88	3750.51	45.15	0.9991
9%PGWA-SBS	−6	343.22	16,117.86	173.50	5992.19	46.96	0.9993
6%PGWA-70#	−6	251.19	12,549.61	122.11	4449.90	49.96	0.9992
9%PGWA-70#	−6	329.32	18,232.28	163.33	6174.83	55.36	0.9991
SBS	−12	233.41	17,053.89	131.05	4295.41	73.06	0.9987
6%PGWA-SBS	−12	318.21	28,329.24	211.24	7185.31	89.03	0.9984
9%PGWA-SBS	−12	730.25	70,419.39	523.41	15,918.83	96.43	0.9982
70#	−12	284.55	21,227.99	160.75	5782.04	74.60	0.9988
6%PGWA-70#	−12	311.83	28,285.42	189.45	5834.55	90.71	0.9981
9%PGWA-70#	−12	564.05	61,644.59	441.57	13,371.32	109.29	0.9982
SBS	−18	460.33	62,151.80	381.32	10,669.07	135.02	0.9974
6%PGWA-SBS	−18	665.39	94,484.06	526.01	12,157.23	142.00	0.9902
9%PGWA-SBS	−18	804.87	153,674.40	820.83	20,295.53	190.93	0.9941
70#	−18	527.73	86,945.83	563.21	16,721.70	164.76	0.9905
6%PGWA-70#	−18	592.53	109,262.90	662.93	20,918.30	184.40	0.9963
9%PGWA-70#	−18	1048.08	210,187.10	1220.75	30,325.67	200.54	0.9928
SBS	−24	1131.68	126,398.00	919.03	12,582.69	111.69	0.9765
6%PGWA-SBS	−24	855.03	122,892.20	914.14	40,672.49	143.73	0.9911
9%PGWA-SBS	−24	1134.47	298,436.20	1642.76	82,232.24	263.06	0.9921
70#	−24	1010.08	300,577.40	1560.10	37,132.83	297.58	0.9958
6%PGWA-70#	−24	928.60	332,167.50	1674.30	54,062.67	357.71	0.9968
9%PGWA-70#	−24	1046.01	436,384.50	2159.79	54,407.05	417.19	0.9948

**Table 3:** Flexibility parameters of PGWA-added asphalt.

Types of Asphalt	Test Temperature (℃)	*J_E_*	*J_V_*	*J_DE_*	*J*_C_
6%PGWA-SBS	−6	5.17 × 10^−3^	2.75 × 10^−2^	1.04 × 10^−2^	5.70 × 10^1^
9%PGWA-SBS	−6	2.91 × 10^−3^	1.49 × 10^−2^	5.76 × 10^−3^	1.06 × 10^2^
6%PGWA-70#	−6	3.98 × 10^−3^	1.91 × 10^−2^	8.18 × 10^−3^	8.55 × 10^1^
9%PGWA-70#	−6	3.04 × 10^−3^	1.32 × 10^−2^	6.11 × 10^−3^	1.29 × 10^2^
SBS	−12	4.28 × 10^−3^	1.41 × 10^−2^	7.63 × 10^−3^	1.31 × 10^2^
6%PGWA-SBS	−12	3.14 × 10^−3^	8.47 × 10^−3^	4.73 × 10^−3^	2.28 × 10^2^
9%PGWA-SBS	−12	1.37 × 10^−3^	3.41 × 10^−3^	1.91 × 10^−3^	5.76 × 10^2^
70#	−12	3.51 × 10^−3^	1.13 × 10^−2^	6.21 × 10^−3^	1.65 × 10^2^
6%PGWA-70#	−12	3.21 × 10^−3^	8.48 × 10^−3^	5.28 × 10^−3^	2.36 × 10^2^
9%PGWA-70#	−12	1.77 × 10^−3^	3.89 × 10^−3^	2.26 × 10^−3^	5.23 × 10^2^
SBS	−18	2.17 × 10^−3^	3.86 × 10^−3^	2.62 × 10^−3^	5.80 × 10^2^
6%PGWA-SBS	−18	1.50 × 10^−3^	2.54 × 10^−3^	1.90 × 10^−3^	9.21 × 10^2^
9%PGWA-SBS	−18	1.24 × 10^−3^	1.56 × 10^−3^	1.22 × 10^−3^	1.65 × 10^3^
70#	−18	1.89 × 10^−3^	2.76 × 10^−3^	1.77 × 10^−3^	8.44 × 10^2^
6%PGWA-70#	−18	1.69 × 10^−3^	2.20 × 10^−3^	1.51 × 10^−3^	1.12 × 10^3^
9%PGWA-70#	−18	9.54 × 10^−4^	1.14 × 10^−3^	8.19 × 10^−4^	2.24 × 10^3^
SBS	−24	8.84 × 10^−4^	1.90 × 10^−3^	1.09 × 10^−3^	1.07 × 10^3^
6%PGWA-SBS	−24	1.17 × 10^−3^	1.95 × 10^−3^	1.09 × 10^−3^	1.10 × 10^3^
9%PGWA-SBS	−24	8.81 × 10^−4^	8.04 × 10^−4^	6.04 × 10^−4^	3.54 × 10^3^
70#	−24	9.90 × 10^−4^	7.98 × 10^−4^	6.41 × 10^−4^	3.81 × 10^3^
6%PGWA-70#	−24	1.08 × 10^−3^	7.23 × 10^−4^	5.97 × 10^−4^	4.59 × 10^3^
9%PGWA-70#	−24	9.56 × 10^−4^	5.50 × 10^−4^	4.63 × 10^−4^	6.51 × 10^3^

## Data Availability

The original contributions presented in this study are included in the article. Further inquiries can be directed to the corresponding author.

## Abbreviations

The following abbreviations are used in this manuscript:

WMA	Warm-mix asphalt
HMA	Hot-mix asphalt
PGWA	Phosphogypsum warm-mix additive
SBS	Styrene–butadiene–styrene
MSCR	Multiple stress creep and recovery
LAS	Linear amplitude sweep
DSR	Dynamic shear rheometer
BBR	Bending beam rheometer
*R*	Creep recovery rate
*J*nr	Non-recoverable compliance
*C*	Integrity index
*S*	Creep stiffness modulus
*m*	Stiffness rate
*E*_1_	Instantaneous elastic modulus
*E*_2_	Delayed elastic modulus
*η*_1_	Instantaneous viscosity coefficient
*η*_2_	Delayed viscosity coefficient
λ	Relaxation coefficient
*J_C_*	Flexibility coefficient
*J_E_*	Instantaneous elastic compliance
*J_V_*	Viscous flow compliance
*J*_DE_	Delayed elastic compliance
